# Supplementary material for: Nrp2 is sufficient to instruct circuit formation of mitral-cells to mediate odour-induced attractive social responses
Source: Nat Commun. 2017 Jul 21;8:15977. doi: 10.1038/ncomms15977 (PMC5525001; doi:10.1038/ncomms15977)
Supplement: Supplementary Information [file ncomms15977-s1.pdf]

Type of file: PDF
Size of file: 0 KB
Title of file for HTML: Supplementary Information
Description: Supplementary Figures

Type of file: PDF
Size of file: 0 KB
Title of file for HTML: Peer Review File
Description:

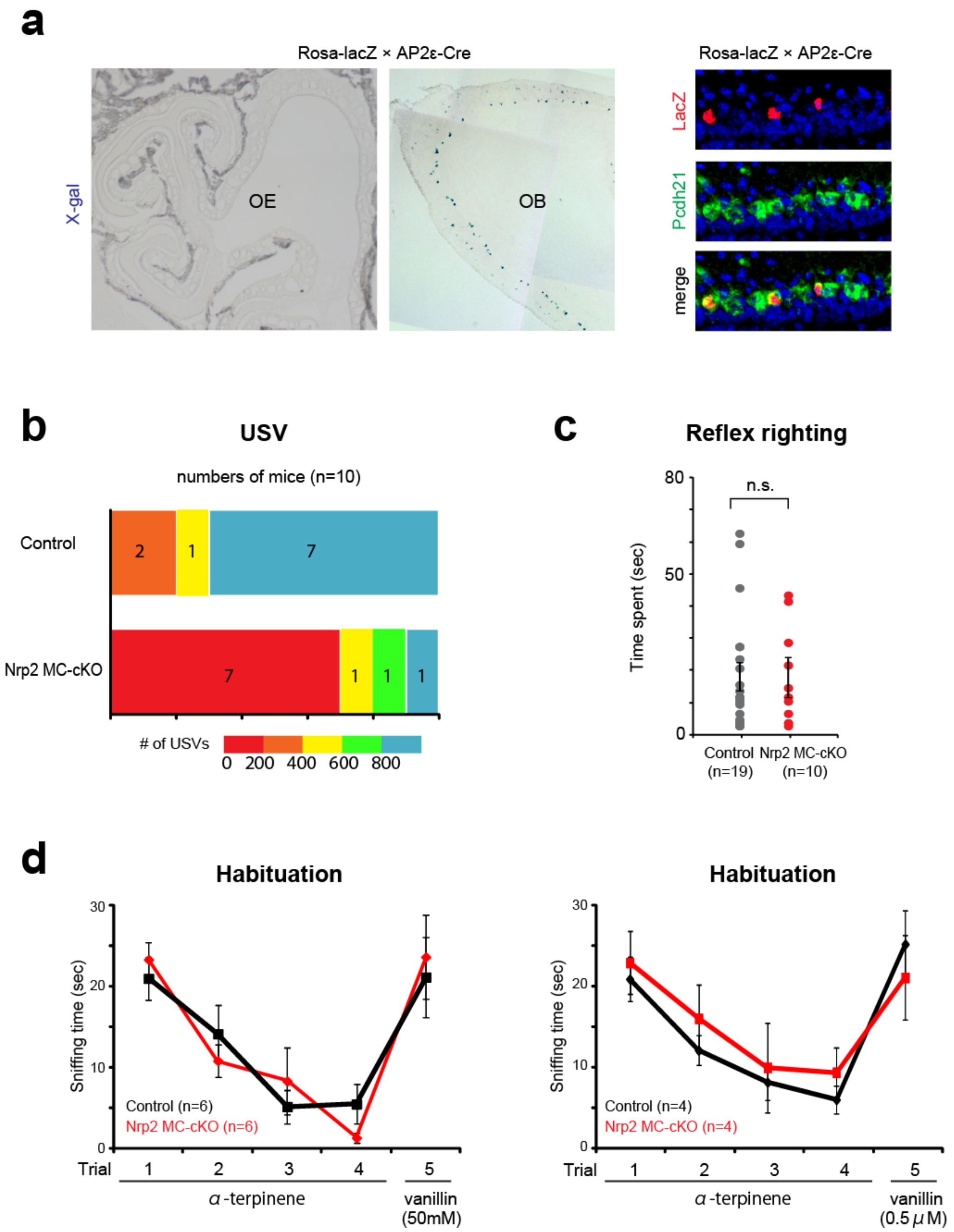
a
Rosa-lacZ × AP2ε-Cre
X-gal
OE
OB
Rosa-lacZ × AP2ε-Cre
LacZ
Pcdh21
merge
b
USV
numbers of mice (n=10)
Control
2
1
7
Nrp2 MC-cKO
7
1
1
1
# of USVs
0 200 400 600 800
c
Reflex righting
80
n.s.
50
0
Time spent (sec)
Control (n=19)
Nrp2 MC-cKO (n=10)
d
Habituation
30
20
10
0
Sniffing time (sec)
Control (n=6)
Nrp2 MC-cKO (n=6)
Trial 1 2 3 4 5
α-terpinene
vanillin (50mM)
Habituation
30
20
10
0
Sniffing time (sec)
Control (n=4)
Nrp2 MC-cKO (n=4)
Trial 1 2 3 4 5
α-terpinene
vanillin (0.5μM)

Supplementary Figure. 1 Odor sensing is not affected by the MC-specific cKO of Nrp2.

(a) Detection of the *AP2ε* promoter activity (left). The Tg AP2ε-Cre mouse was crossed with the ROSA-shutter-lacZ mouse for Cre-mediated *lacZ* induction. Coronal sections of the OE and a parasagittal section of the OB at P14 were analyzed by X-gal staining. MCs were stained blue in the OB. $LacZ^+$ $PCdh21^+$ cells in the MCL (right). Parasagittal OB section were immunostained with antibodies against β-gal and Pcdh21. **(b)** Distribution of numbers of male USV. USV was counted in the presence of a female intruder for duration of 6 min in each experiment. (**c**) Locomotor abilities test. Mice were placed on their back. The time they spent to recover from this position, to being upright and on four paws, was measured (reflex righting). No difference was found between the cKO and heterozygous littermates in their recovery time lengths. **(d)** Habituation-dishabituation test. In this test, α-terpinene was presented in four consecutive trials for duration of 1 min. The inter-trial interval was 10 min. Then, a novel odor vanillin (50 mM or 0.5 μM) was presented. Habituation is defined by a progressive decrease in sniffing towards the repeated presentation of the same odor stimulus. Dishabituation is defined by reinstatement of sniffing when the novel odor is presented. Graphs demonstrate amounts of time that the male cKO and control littermates spend on sniffing a piece of filter paper spotted with vanillin or α-terpinene. Trials with vanillin as the repetitive odor and α-terpinene as the novel odor yielded similar results. No difference is found between the cKO and control mice. Error bars are ±SE. Error bars are ±SE. n.s.: not significant (Student's *t* test).

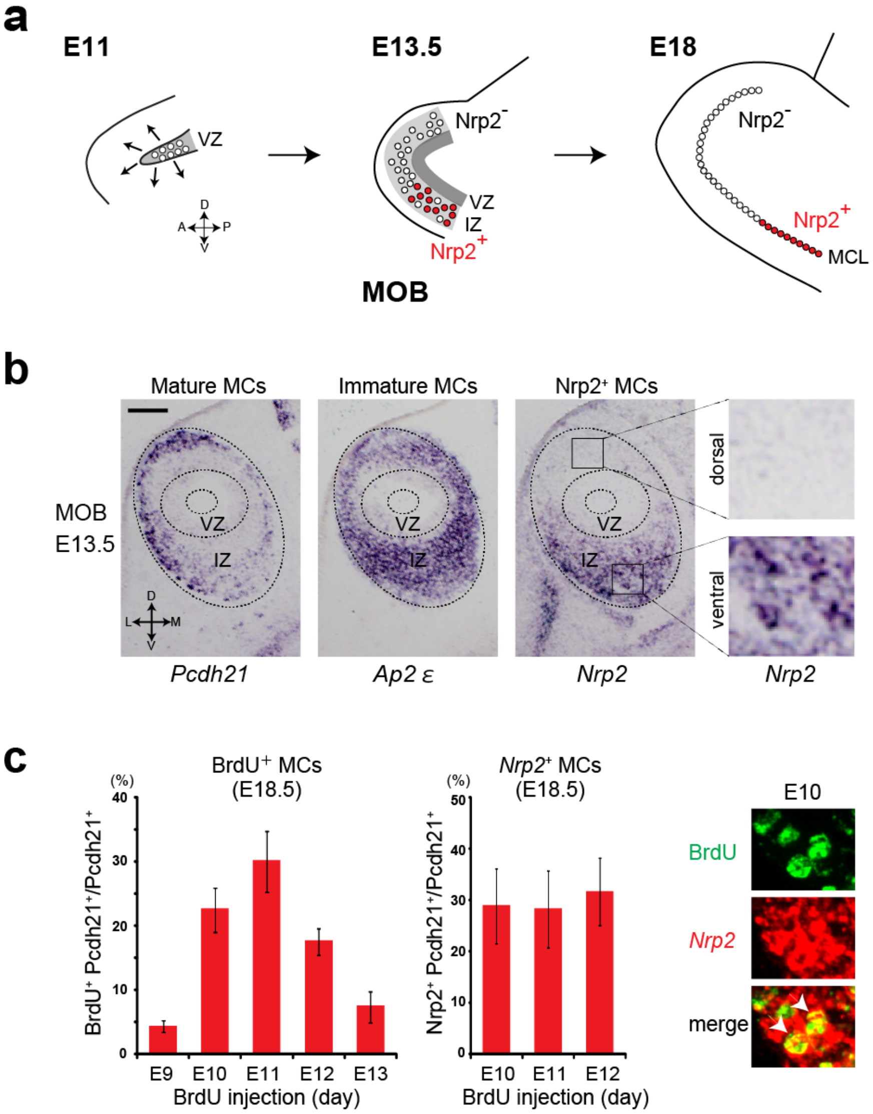

Supplementary Figure. 2 Developmental expression of *Nrp2*.

(a) Distribution of *Nrp2*$^+$ mitral cells (MCs) in the embryonic MOB. Serial MOB sections at E13.5 were hybridized with the *Nrp2*, *AP2ε* (immature MC marker) and *Pcdh21* (mature MC marker) probes. During development, MCs are generated in the ventricular zone (VZ) and migrate radially to the MOB surface through the intermediate zone (IZ). **(b)** Generation of *Nrp2*$^+$ MCs during embryonic development. BrdU was injected into pregnant mice at E9, 10, 11, 12 and 13. At E18.5, MOB sections were immunostained with anti-BrdU antibodies and hybridized with the *Nrp2* probe. Ratios (%) of MCs labeled with BrdU at indicated time points

are shown (left). Percentages of *Nrp2*$^+$ MCs labeled with BrdU are compared at E10, 11 and 12 (middle). BrdU$^+$ and *Nrp2*$^+$ MCs injected at E10 are shown in the right. A scale bar is 200 µm. Data are presented in mean ± SE. **(c)** Schematic diagrams of MC migration during development. In the embryo, MC precursors are generated in the VZ and migrate radially to the surface of MOB (left). Nrp2$^+$ and Nrp2$^-$ immature MCs are segregated in the IZ (middle). Nrp2$^+$ and Nrp2$^-$ MCs are distributed in the posteroventral and dorsal MCL, respectively. VZ, ventricular zone; IZ, intermediate zone; MCL, mitral cell layer.

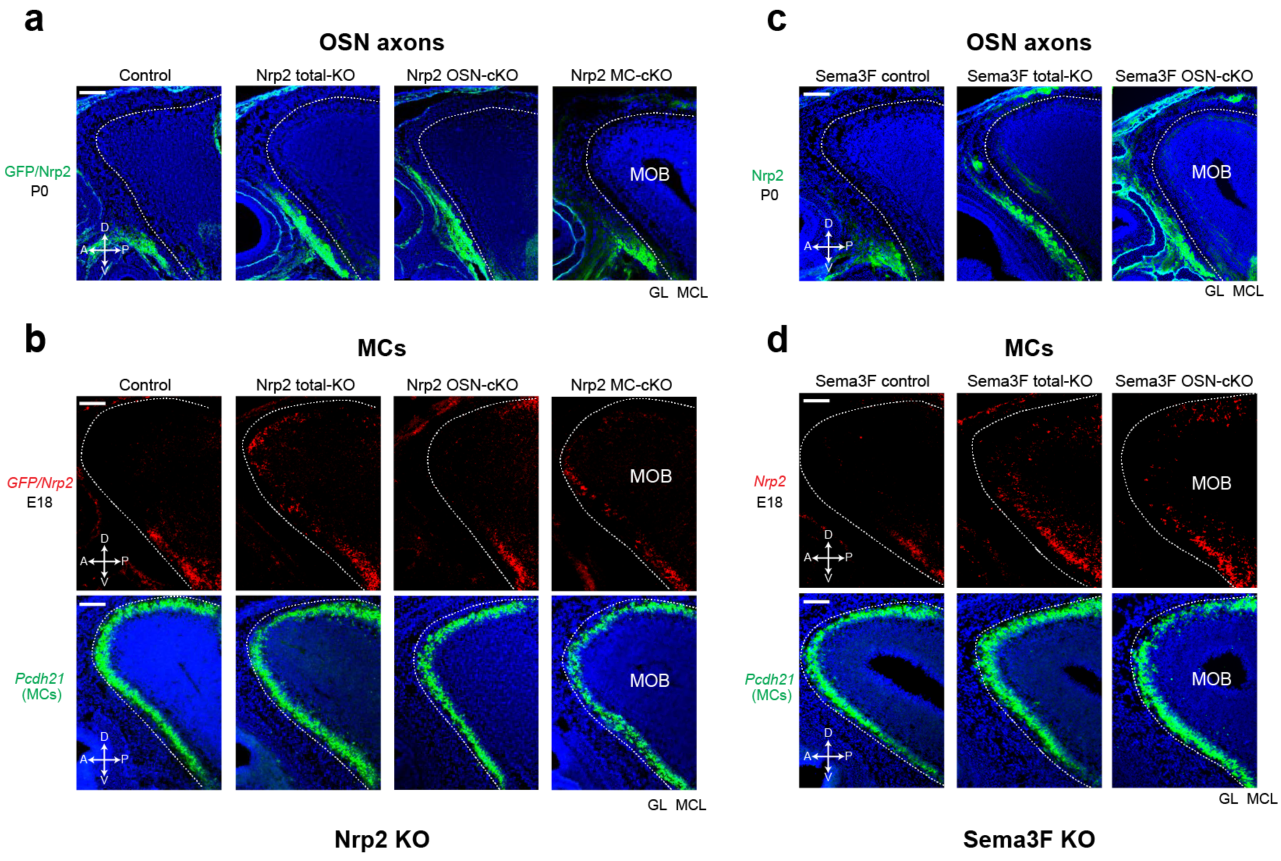

Supplementary Figure. 3 Nrp2-Sema3F repulsive interactions regulate both OSN projection and MC distribution in the MOB.

(a) Targeting of V-zone OSN axons in the Nrp2 KOs. To detect ventral OSN axons that normally express Nrp2, the *Nrp2* locus was replaced with the *GFP* in the KO allele. Parasagittal MOB sections at P0 were immunostained with anti-GFP antibodies. For the MC specific KO of Nrp2 (Nrp2 MC cKO), MOB sections were immunostained with anti-Nrp2 antibodies. **(b)** Distribution of *Nrp2*$^{+}$ MCs in the MOB in the Nrp2 KOs. Parasagittal sections at E18 were analyzed by *in situ* hybridization using probes for *GFP* and *Nrp2*, and *Pcdh21* (MC marker). **(c)** Targeting of V-zone OSN axons in the Sema3F KOs. Parasagittal MOB sections at P0 were immunostained with anti-Nrp2 antibodies. **(d)** Distribution of *Nrp2*$^{+}$ MCs in the MOB in the

Sema3F KOs. Parasagittal MOB sections at E18 were analyzed by *in situ* hybridization using probes for *Nrp2* and *Pcdh21* (MC marker). In all figures, broken lines indicate the boundary of the glomerular layer (GL) and mitral-cell layer (MCL) in the MOB. Scale bars are 200 μm.

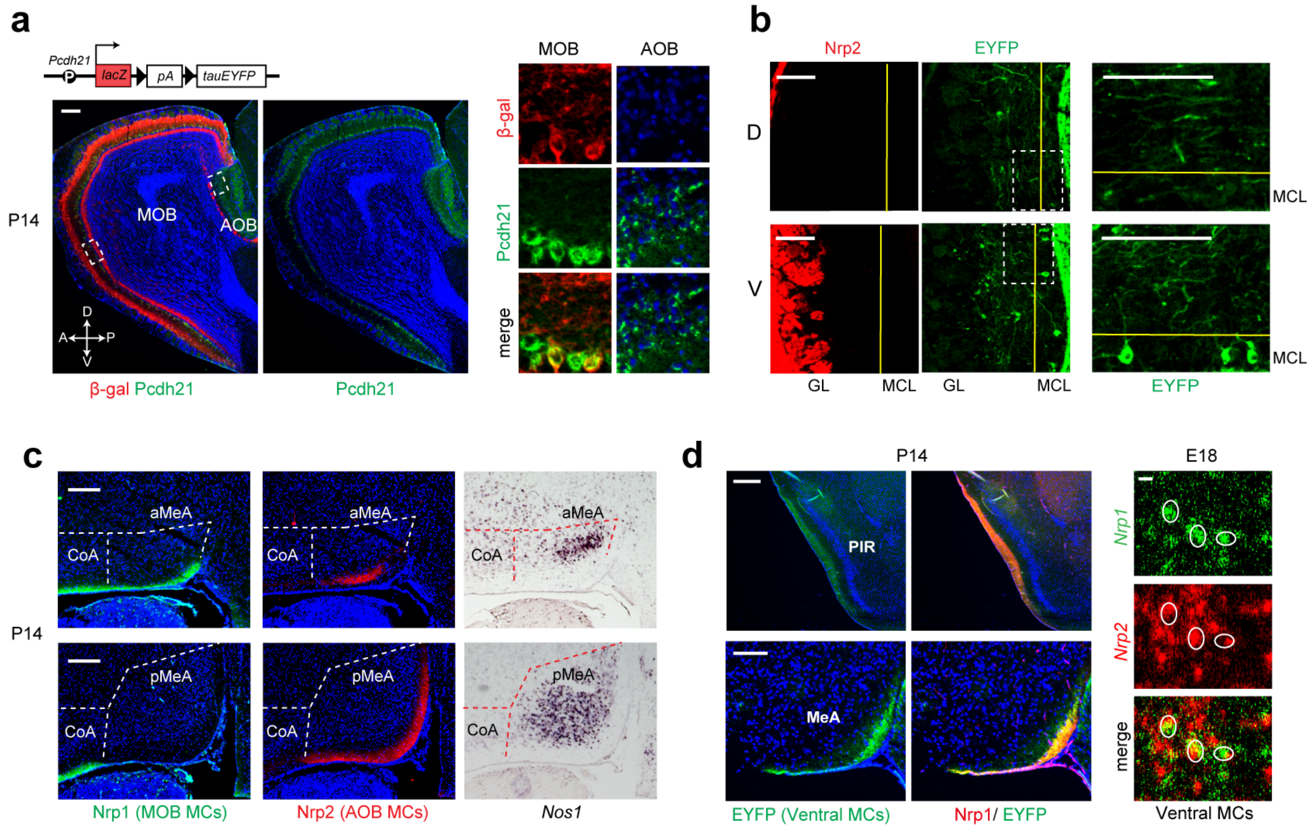

Supplementary Figure. 4 Labeling of *Nrp2*$^{+}$ MCs in the MOB.

(a) MC-specific activation of the *Pcdh21* promoter in the MOB. A parasagittal section at P14 was immunostained with antibodies against β-galactosidase (β-gal; red) and Pcdh21 (MC marker; green) (left). Enlarged photos of the boxed regions are shown in the right. Note that β-gal signals are detected in the MOB MCs, but not in the accessory olfactory bulb (AOB). This mouse line is quite useful for the analysis of MOB MCs independent of AOB counterparts for axonal projection. **(b)** V-region-specific activation of the *Nrp2* promoter in the MCL. Nrp2-Cre mouse was crossed with the Tg mouse Pcdh21-lacZ-STOP-tauEYFP to induce EYFP

specifically in the MOB MCs. A coronal MOB section at P14 was immunostained with antibodies against GFP. EYFP-positive MCs (green) are found in the ventral (V), but not in the dorsal (D) region of the MCL. **(c)** Nrp2^{+} MOB MCs send their axons to the anterior MeA but not to the posterior MeA. Coronal sections of the OC at P14 were immunostained with anti-Nrp1 and anti-Nrp2 antibodies to detect MOB- and AOB-MC axons, respectively. Note that Nrp2 is not expressed in MOB MCs at this stage (P14). Thus, Nrp2 can be used as an AOB-MC marker. Serial OC sections were also analyzed by *in situ* hybridization using the *Nos1* probe, a marker for MeA. **(d)** Detection of Nrp1 (MOB MC marker) in the *Nrp2*$^{+}$ (EYFP^{+}) ventral MC axons (green, left column). Coronal sections of the OC at P14 were immunostained with anti-GFP and anti-Nrp1 antibodies. GFP^{+} cells are all positive for Nrp1. Scale bars are 100 μm in (a-c & d left) and 10 μm in (d right).

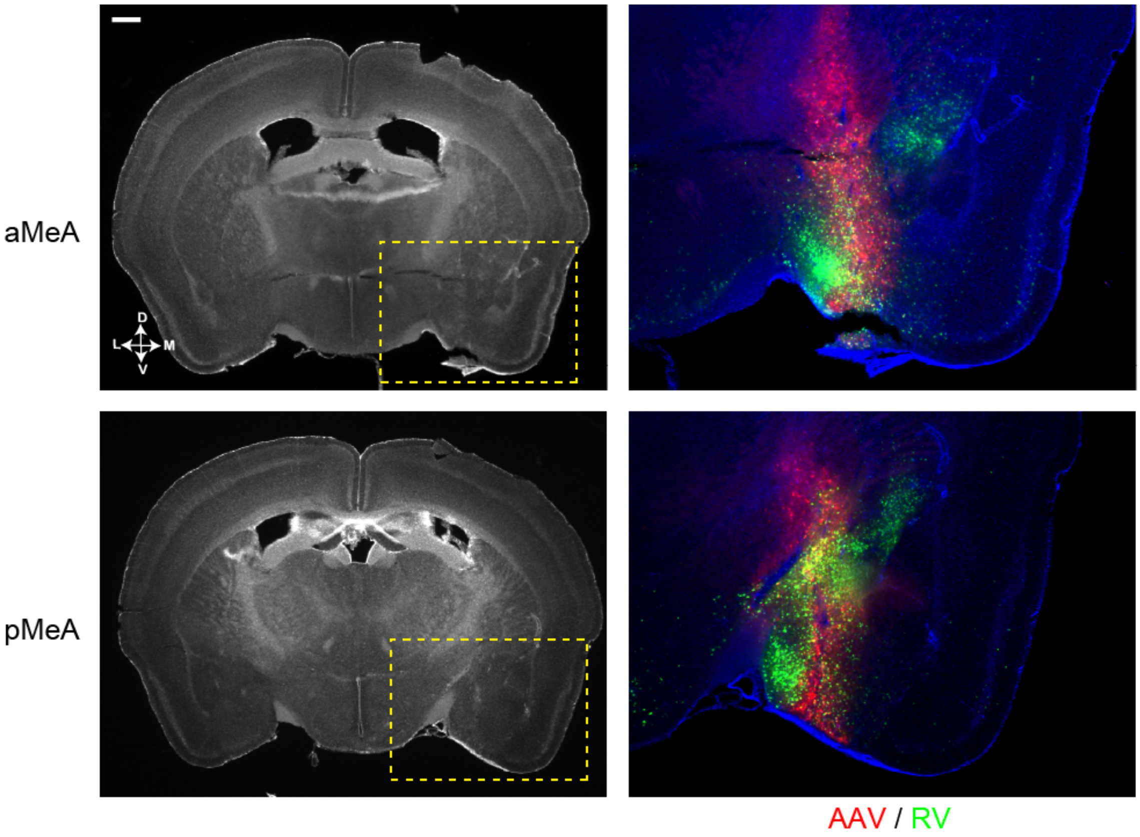

Supplementary Figure. 5 Virus injection into the MeA.

AAV was first injected into the MeA region to complement TVA and glycoprotein G. After 2 weeks, the RV was injected into the same MeA region to selectively infect neurons expressing the TVA receptor. After 10 days, serial coronal sections of the entire MeA were analyzed to determine whether the neurons infected with AAV and RV were indeed confined to the MeA. Double-labeled cells with mCherry for AAV (red) and EGFP for RV (green) are detected in the MeA. Scale bar is 100 μm.

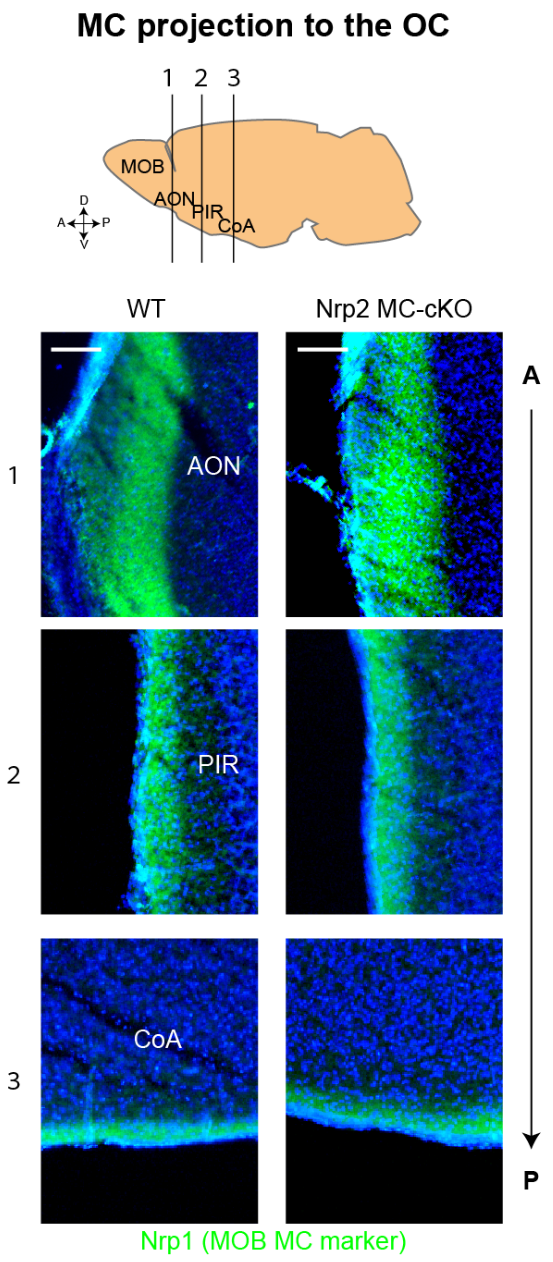

Supplementary Figure. 6 Projection of MOB MCs to the OC.

Detection of MC axons in the OC. Coronal section of the OC at P14 were immunostained with antibodies against Nrp1 (MOB MC marker). Locations of OC sections are indicated at the top. Scale bars are 100 μm.

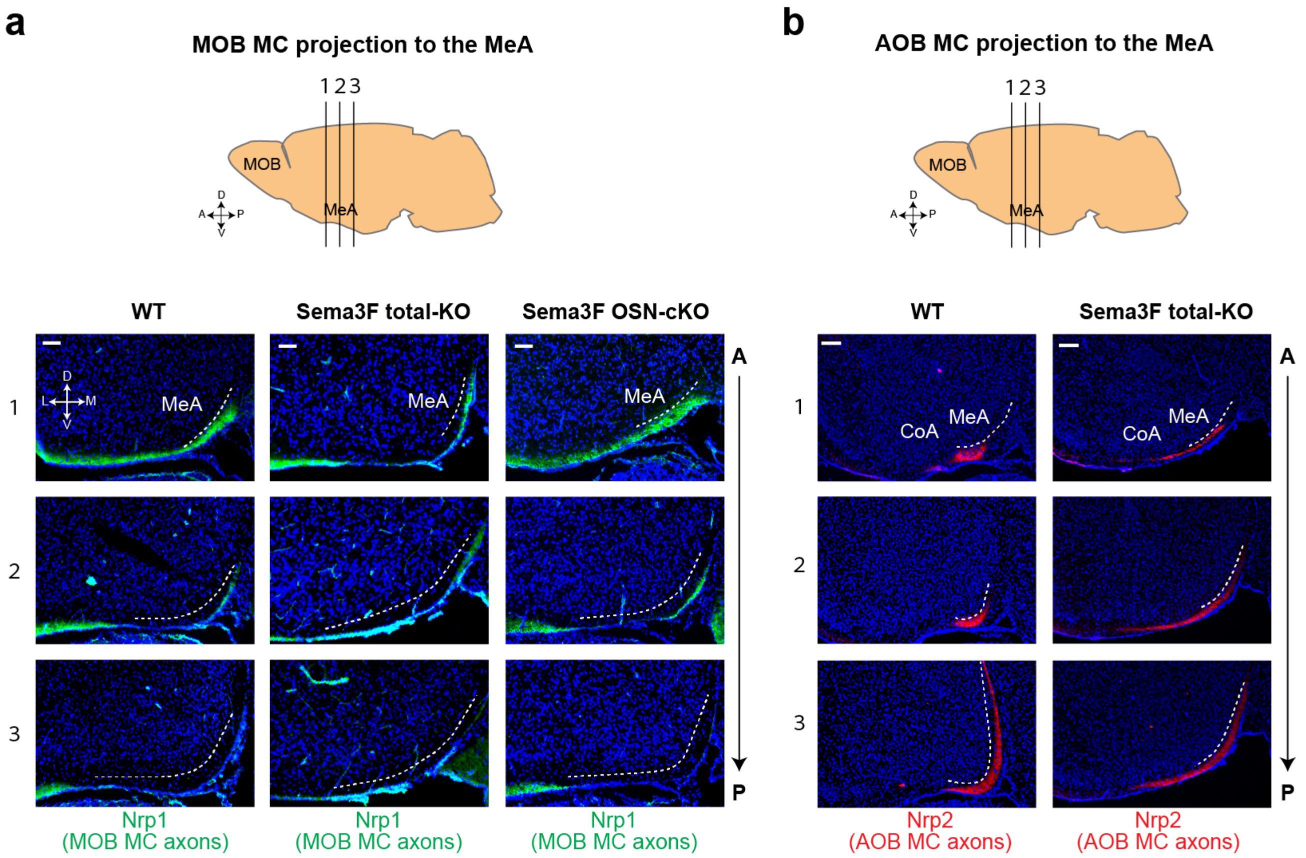

Supplementary Figure. 7 MC projection to the MeA.

(a) MOB MCs. Serial coronal sections (1~3) of the OC (each separated by 200 μm) at P14 were immunostained with antibodies against Nrp1 to detect MOB MC axons. $Nrp1^{+}$ axons (green) are defasciculated in the MeA region of the Sema3F total KO. As in the WT, Nrp1 signals are detected in the MeA in the OSN-specific Sema3F KO (OSN cKO). **(b)** AOB MCs. Serial coronal sections (1~3) of the OC (each separated by 160 μm) at P14 were immunostained with antibodies against Nrp2 to detect AOB MC axons. Note that at this stage (P14), Nrp2 is not expressed in MOB MC axons. Thus, Nrp2 can be used as an AOB-MC marker. $Nrp2^{+}$ axons (red) are defasciculated in the MeA region of the Sema3F total KO. Broken lines in the sections indicate the MeA. Scale bars are 100 μm.

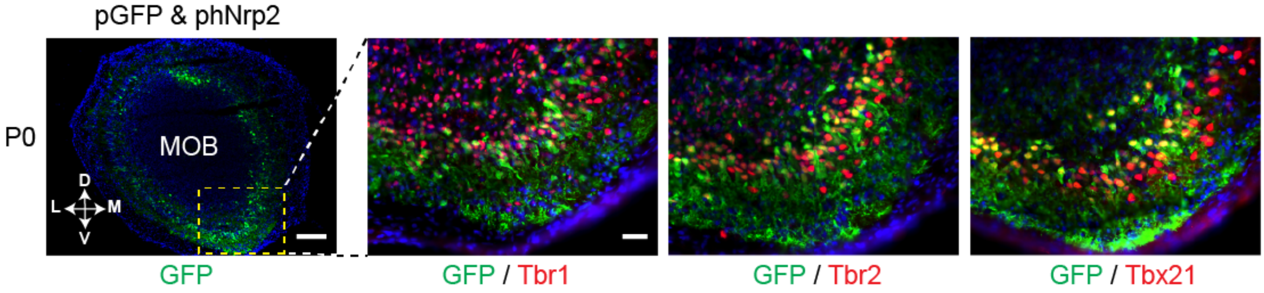

Supplementary Figure. 8 ***In utero*** **electroporation in the embryonic OB.**

Plasmid vectors containing the *EGFP* (pGFP) with or without human *Nrp2* cDNA (phNrp2) were electroporated into the WT embryonic MOB at E11. Coronal MOB sections at P0 were isolated and immunostained with antibodies against GFP, Tbr1, Tbr2, and Tbx21 (MC markers). A fraction of MCs ectopically expressed EGFP. Scale bars are 100 µm (left) and 20 µm (right).

In all extended data, dimensions are: D, dorsal; V, ventral; A, anterior; P, posterior; L, lateral; M, medial.
